# Changes in gut microbiota composition following water kefir consumption in healthy adults

**DOI:** 10.1038/s41598-026-53645-7

**Published:** 2026-05-22

**Authors:** Rumathi de Mel, Alia H. Al Khafaji, Saraladevi Muthusamy, Jie Xu, Åsa Håkansson

**Affiliations:** 1 Department of Process and Life Science Engineering, Faculty of Engineering, Lund University, Lund, Sweden; 2 Scientific Department, Warith International Cancer Institute, Karbala, Iraq

**Keywords:** Water kefir, Fermentation, Plant-based alternatives, Probiotic potential, Gut health, Next generation sequencing, Diversity, Relative abundance, Gastroenterology, Microbiology

## Abstract

Fermented foods have gained increasing scientific interest for their potential to modulate gut microbiota and provide probiotic microorganisms with possible health benefits. This intervention trial examined the impact of daily consumption of water kefir, a sustainable plant-based fermented beverage, on gut microbiota composition in 40 healthy adults. Participants consumed 200 mL of homemade water kefir daily for 14 days, with fecal samples collected before and after the intervention. Some participants reported mild, transient gastrointestinal effects such as flatulence (32%) and bloating (24%), which are common when introducing live microorganisms, while others experienced reduced abdominal pain (28%), and most (66%) reported no noticeable change in symptoms. 16S rRNA sequencing revealed significant shifts in microbial composition, including a 6.5% decrease in Firmicutes and increases in Bacteroidetes (+ 21.6%) and Actinobacteria (+ 14.8%). At the species level, beneficial taxa such as *Blautia* spp. and *Roseburia faecis* increased, along with commensals including *Bacteroides fragilis*, *Bacteroides uniformis*, *Gemmiger formicilis*, *Prevotella copri*, and *Parabacteroides distasonis* (*p* < 0.01). Although *α-*diversity remained unchanged, *β*-diversity differed significantly between pre- and post-intervention samples (*p* = 0.025). By comparing the relative abundance of dominant genera in participants’ gut microbiota and in water kefir, overlapping genera such as *Lactobacillus*, *Bifidobacterium*, *Prevotella*, *Coprococcus*, and *Faecalibacterium* were identified. Among these, *Bifidobacterium* and *Prevotella* increased, *Coprococcus* decreased, and *Lactobacillus* and *Faecalibacterium* remained stable. Genera exclusive to the gut microbiota also exhibited differential changes. These findings suggest that water kefir consumption is associated with modulation of the gut microbiota, including increases in saccharolytic and short-chain fatty acid (SCFA)-producing taxa, potentially influenced by its exopolysaccharides and microbial community. Some genera from water kefir may transiently affect the gut microbiome, and the concurrent increase in *Bifidobacterium* and *Prevotella* may suggest a potential probiotic-like effect. However, causality cannot be established, and further studies are needed to assess the persistence of these changes and their long-term clinical relevance.

## Introduction

Fermented foods and beverages have been a part of human diets for centuries^1^. According to the International Scientific Association for Probiotics and Prebiotics (ISAPP), fermented foods are defined as “Foods made through desired microbial growth and enzymatic conversions of food components”^2^. Probiotics, by contrast, are defined as “Live microorganisms that, when administered in adequate amounts, confer a health benefit on the host”^3^. While probiotic strains are specific microorganisms with demonstrated health benefits, fermented foods result from microbial activity but may not necessarily contain live microorganisms with probiotic properties^4^. Despite this distinction, both have attracted increasing attention due to their potential health effects. This growing interest is supported by emerging evidence highlighting the benefits of fermented foods^5^.

Among these, kefir has gained attention as a widely consumed functional beverage. Water kefir, the non-dairy kefir variety, is an acidic and slightly alcoholic beverage produced through fermentation of small kefir grains with water. Water kefir grains (also known as tibicos) consist of a symbiotic starter culture of lactic acid bacteria and yeasts, some of which are considered probiotic, embedded in a complex matrix of polysaccharides and proteins. The matrix is primarily composed of dextran, an exopolysaccharide produced by certain strains of *Lactobacillus* (e.g., *Lactobacillus hilgardii*), which forms the gelatinous structure of the grains. This matrix also includes structural proteins and other microbial byproducts that support the stability and cohesion of the grain community. Sucrose, fruits, and berries may be incorporated into the fermentation process as a source of fermentable sugars or to contribute to the unique flavour profile of the final product. These include, for example, dried apricots, figs, pomegranates, and lemons^6^.

For industrial production of water kefir, three key stages are generally followed: the initial fermentation process, a rest phase at lower temperatures, and the final fermentation process. After completion of the final process, water kefir grains are separated from the liquid by sieving. Fermentation temperatures are maintained between 25 °C and 30 °C, which results in a pH drop from 7.0 to 3.0-4.5. The primary microorganisms that grow during water kefir production are yeast species like *Saccharomyces cerevisiae* and lactic acid bacterial species like *Lactobacillus nagelii*,* Lactobacillus hilgardii* and *Lactobacillus paracasei*^7^. The water kefir beverage produced in the fermentation process also contains various microbial metabolites, including short-chain fatty acids (SCFAs), vitamins, and bioactive compounds^8^.

Despite the long-standing tradition of consuming fermented foods, the potential health effects of water kefir remain underexplored. To date, research has been limited and primarily based on experimental models, although its diverse microbial community suggests a possible probiotic potential. Calatayud et al. (2021) evaluated water kefir products in an in vitro model simulating colonic fermentation and the intestinal epithelium. They observed increased SCFA production, reduced levels of detrimental proteolytic fermentation compounds, and an enrichment of *Bifidobacterium* spp. Animal studies have similarly reported favourable effects on plasma and hepatic lipid profiles^9^.

In contrast, dairy kefir has been extensively studied in numerous clinical trials, demonstrating benefits such as immunomodulation, antimicrobial activity, and gut microbiome modulation^10^. Moreover, clinical studies have demonstrated that dairy kefir can improve lactose digestion and tolerance in individuals with lactose maldigestion, suggesting its functional potential beyond basic nutrition^11^. These findings highlight kefir’s capacity to influence host physiology and gut function, reinforcing the need to evaluate whether beneficial effects occur with its non-dairy counterpart, water kefir. As a plant-based alternative to dairy kefir, water kefir also aligns with the growing interest in sustainable, lactose-free, and vegan fermented foods. However, no human intervention studies have yet examined its effects on the gut microbiota or confirmed its probiotic potential in vivo, underscoring the need for evaluation in human subjects.

In the present trial, the effects of daily water kefir intake in 40 healthy adults were investigated. Fecal samples collected before and after the intervention were analysed using next generation sequencing to assess changes in gut microbiota composition. In addition, the microbial profile of the water kefir beverage was compared to that of the participants’ fecal samples to investigate whether kefir-associated microbes colonized the gut or exerted a transient impact on the microbial community.

## Materials and methods

### Enrolment of study participants

Eligible participants represented diverse national backgrounds; however, all individuals were residing in Lund, Sweden, prior to the start of the study in November 2022 and remained there for the duration of the study, which may have reduced variability related to environmental exposure and dietary patterns. Participants were aged between 18 and 65 years and were required to have access to digital devices to complete forms and questionnaires electronically.

Individuals with diagnosed gastrointestinal or autoimmune diseases, or those undergoing medical treatment resulting in immunodeficiency (e.g., multiple sclerosis, type 1 diabetes, cancer, or HIV), were excluded. To minimize potential confounding effects on gut microbiota composition, participants were required to refrain from antibiotic use for at least one month prior to and throughout the study period.

**Fig. 1:**
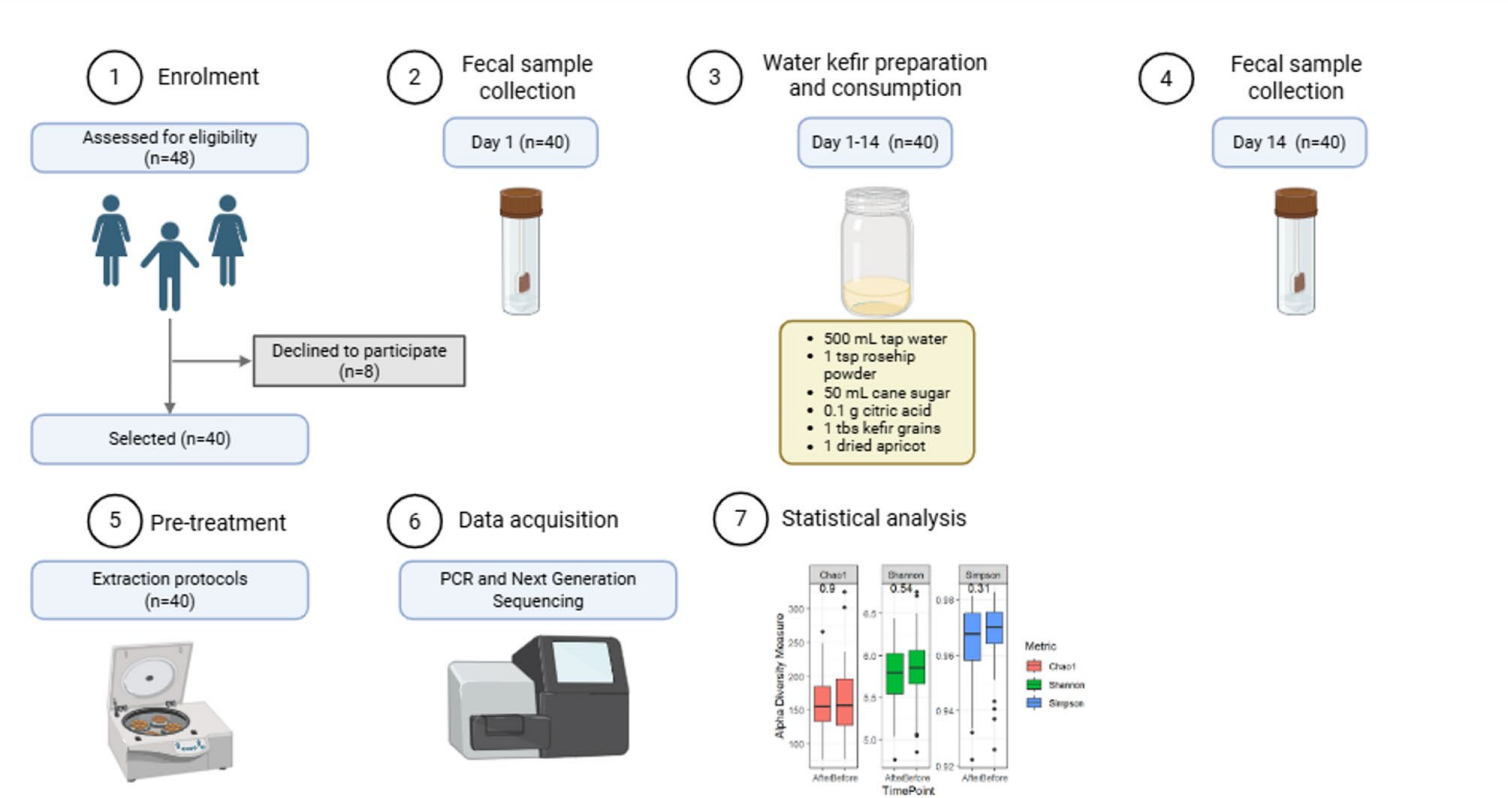
Overview of the study design and methodology for assessing changes in gut microbiota composition following water kefir consumption in healthy adults. A total of 40 individuals were included in the final analysis. Fecal samples were self-collected by participants on two occasions (before and at the end of consumption) and used for DNA extraction and 16S rRNA gene amplicon sequencing. During the intervention, participants consumed a daily preparation of water kefir. Sequencing was performed using the Illumina MiSeq platform. The obtained sequences were used to assess whether water kefir consumption could modulate gut microbiota and promote the presence of potentially beneficial microorganisms. Illustration created in https://BioRender.com.

A total of 48 eligible participants (36 females and 12 males) provided written informed consent in accordance with the Declaration of Helsinki. The study was approved by the Swedish Ethical Review Authority (Dnr 2022-05369-01) and registered at ClinicalTrials.gov (NCT05657730; first registered on 09/11/2022). This study was designed as an exploratory single-arm pre–post intervention trial. The overall study design is illustrated in Fig. 1.

### Water kefir preparation and consumption

Participants prepared their water kefir beverages at home using ingredients provided by the Department of Process and Life Science Engineering, Faculty of Engineering, Lund University, Sweden. To ensure consistency, all ingredients and equipment were newly purchased and provided to participants, and standardized protocols were implemented through detailed written instructions, video tutorials, and oral guidance. Prior to preparation, participants adhered to strict hand hygiene protocols and ensured that all equipment was thoroughly cleaned with detergent. Water kefir grains (Gryningen Hälsa AB, Stockholm, Sweden) were rinsed with cold tap water prior to use, and the rinse water was discarded.

To prepare the beverage, 500 mL of cold tap water was added to a clean glass jar, along with one teaspoon of organic rosehip powder (KoRo Handels GmbH, Berlin, Germany), 50 mL of raw granulated cane sugar (Dansukker, Nordic Sugar AB, Arlöv, Sweden), citric acid (0.2 g/L) (Santa Maria AB, Mölndal, Sweden), and one whole organic dried apricot (Saltå Kvarn AB, Järna, Sweden). The dried apricot was included to support fermentation and enhance the flavor profile and was removed prior to consumption; participants were instructed not to consume the fruit. The mixture was gently rotated to ensure even mixing, followed by the addition of one tablespoon of rinsed water kefir grains. The jar was covered with cheesecloth secured with a rubber band and left to ferment at room temperature for 48 h.

After fermentation, the water kefir was stored under refrigeration until consumption. A batch of 500 mL was sufficient for two days, and participants were instructed to prepare a new batch every second day to ensure a continuous supply throughout the two-week intervention. Participants consumed 200 mL of the fermented water kefir beverage daily and were instructed to maintain their habitual diet throughout the study while avoiding all commercial probiotic products and fermented foods during the two-week washout period prior to enrolment and throughout the intervention.

Gastrointestinal well-being and digestive symptoms were monitored using an online exploratory questionnaire. No detailed dietary intake data were recorded.

### Fecal sample collection

Fecal samples were self-collected by participants on two occasions: either on the day before or the same day as initiating water kefir consumption (prior to the first intake), and on the final day of consumption or the following day (after 14 days). Pre-labelled fecal collection tubes (76 × 20 mm; Sarstedt, Sweden) were provided by the Department of Process and Life Science Engineering, Lund University. Participants were instructed on proper sample collection procedures.

After collection, samples were stored at − 20 °C by participants until delivery to the Department, where they were subsequently stored at − 80 °C until analysis.

### Assessment of gastrointestinal symptoms

Gastrointestinal well-being and digestive symptoms were assessed using an online questionnaire developed in Webropol (Webropol Oy, Finland). The questionnaire was adapted from previously validated surveys used in dietitian and consumer studies related to functional foods^12–14^ and was approved by the Swedish Ethical Review Authority (Dnr 2022-05369-01); however, it was not a formally validated clinical instrument.

Participants completed the questionnaire before and after the intervention period. Self-reported symptoms included bloating, flatulence, stomach pain, and general gastrointestinal discomfort.

The survey consisted of both categorical (e.g., presence or absence of symptoms) and Likert-type scale questions to assess the frequency and severity of symptoms. Responses were collected electronically and used for descriptive analysis of gastrointestinal outcomes.

### DNA extraction

DNeasy PowerSoil Pro Kit (Qiagen, Venlo, Netherlands) was used to extract bacterial DNA from fecal samples, according to the manufacturer’s instructions. Briefly, 0.25 g fecal sample was added to PowerBead Pro tubes and lysed in 800 µL CD1 solution, followed by vortexing and centrifugation. The resulting supernatant (500 µL) was treated with 200 µL of CD2 solution, followed by vortexing and centrifugation. 600 µL of CD3 solution was added to the resulting supernatant, mixed, and loaded onto a MB Spin Column for centrifugation. This was followed by washing steps using EA and C5 solutions and DNA elution with C6 solution. The final DNA concentration was determined using NanoDrop spectrophotometer (Thermo Scientific, MA, USA).

### Next generation sequencing

The V3–V4 hypervariable region of the 16S rRNA gene was amplified according to the 16S metagenomic sequencing library preparation protocol provided by Illumina^15^. The primer pairs 341F (5′-TCG TCG GCA GCG TCA GAT GTG TAT AAG AGA CAG CCT ACG GGN GGC WGC AG-3′) and 805R (5′-GTC TCG TGG GCT CGG AGA TGT GTA TAA GAG ACA GGA CTA CHV GGG TAT CTA ATC C-3′) (Eurofins Genomics, Ebersberg, Germany) were used with PCR reagents Kapa HiFi Hotstart Ready Mix (Kapa Biosystems, South Africa).

The PCR program included an initial denaturation step at 95 °C for 3 min, followed by 25 cycles of denaturation at 95 °C for 30 s, annealing at 55 °C for 30 s, extension at 72 °C for 30 s, and a final extension step at 72 °C for 5 min. PCR products were purified using AMPure XP beads (Beckman Coulter Genomics, Brea, CA, USA).

Next, an index PCR was performed using forward adaptors (5′-CAAGCAGAAGACGGCATACGAGAT[i7]GTCTCGTGGGCTCGG-3′) and reverse adaptors (5′-AATGATACGGCGACCACCGAGATCTACAC[i5]TCGTCGGCAGCGTC-3′) from the NexteraXT Index Kit v2 (Illumina, San Diego, CA, USA). The index PCR conditions included an initial denaturation at 95 °C for 3 min, followed by 8 cycles of denaturation at 95 °C for 30 s, annealing at 55 °C for 30 s, extension at 72 °C for 30 s, and a final extension step at 72 °C for 5 min.

The indexed samples were diluted to 4 nM with resuspension buffer (Illumina, San Diego, CA, USA) and sequenced on Illumina Miseq with Miseq reagent kit v3 (600-cycle) (Illumina Inc., San Diego, CA, USA) with a read length of 2 × 300 bp paired-end sequencing according to the manufacturer’s instructions. PhiX Control v3 (Illumina, San Diego, CA, USA) was used as an internal control. The final loading volume was 600 µL.

### Calculations and statistical analysis

The raw sequence data was analysed using QIIME2, version 2022.8^16^. Initially, primers were trimmed using the qiime cutadapt trim-paired plugin^17^. The sequences were denoised using DADA2 to produce representative sequences and feature tables. Taxonomic classification was performed using a pre-trained classifier targeting the V3-V4 region of the 16S rRNA gene^18^. Taxonomic filtering was applied to exclude archaea, chloroplast, and mitochondrial sequences, retaining bacterial sequences for further analysis. *α* and *β* diversity metrics were calculated based on rarefied data at an appropriate sequencing depth determined from the rarefaction curve. *α*-diversity was assessed using Shannon, Chao1, and Simpson’s diversity indices, while *β*-diversity was calculated with the Bray-Curtis distance matrix, and statistical significance was evaluated through ANOSIM.

Differential abundance between groups was analysed at the phylum and species level using DESeq2^19^, with a *p-*value threshold of 0.01. All calculations and statistical analyses were conducted in R version 4.4.0^20^ using the qiime2R, vegan, and ggplot2 packages. No formal sample size or power calculation was performed, as the study was designed as an exploratory investigation.

## Results

### Intervention adherence and perceived effects

Of the 48 participants enrolled in the study, 8 did not provide fecal samples. Consequently, 40 participants (31 females and 9 males) were included in the final analysis. In addition, 38 participants completed the online questionnaire (30 females and 8 males).

Gastrointestinal responses were assessed using a self-reported online questionnaire. After 14 days of water kefir consumption, responses varied among participants. Increased flatulence was reported by 32% of participants, while 24% experienced increased bloating. In contrast, 28% reported a reduction in abdominal pain. The majority of participants (66%) reported no noticeable change in gastrointestinal symptoms. A small proportion (3%) reported transient stomach pain at some point during the study, whereas 97% did not report this symptom.

### Qualitative and quantitative assay of fecal samples

#### Relative abundance

**Fig. 2:**
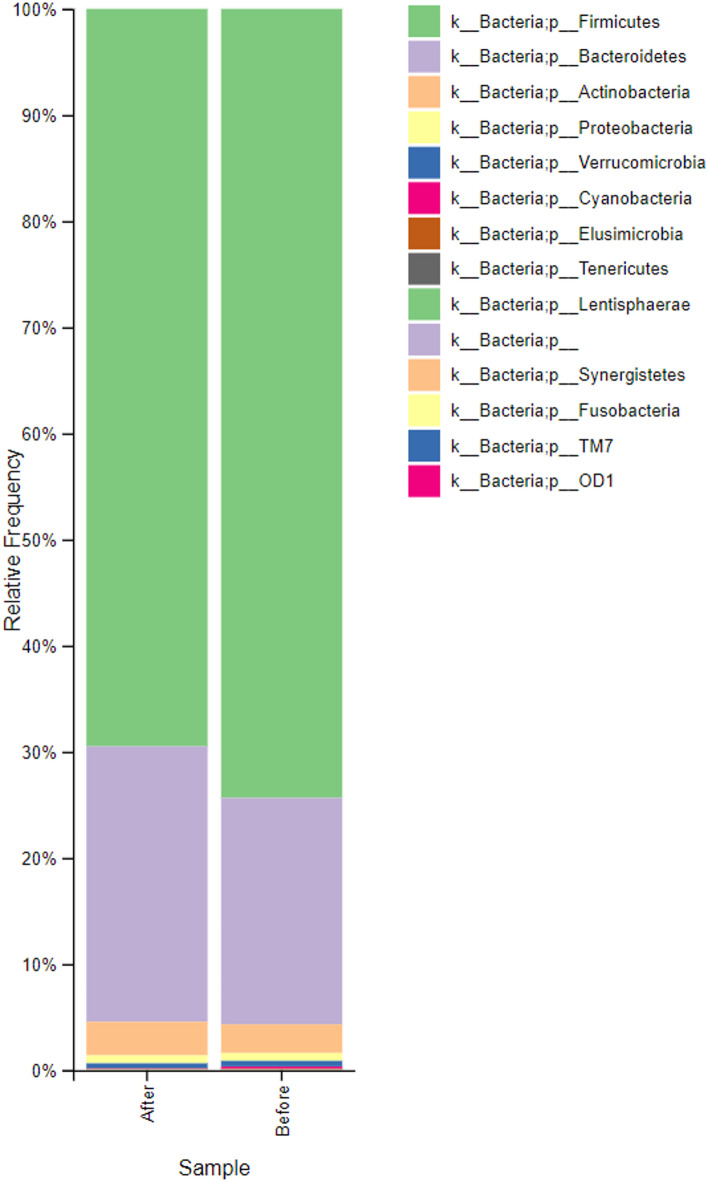
Relative bacterial abundance at the phylum level in 40 fecal samples collected before and after water kefir consumption. All pre-consumption and post-consumption samples were aggregated and presented as summary bars to enhance clarity and facilitate interpretation of the data.

**Fig. 3:**
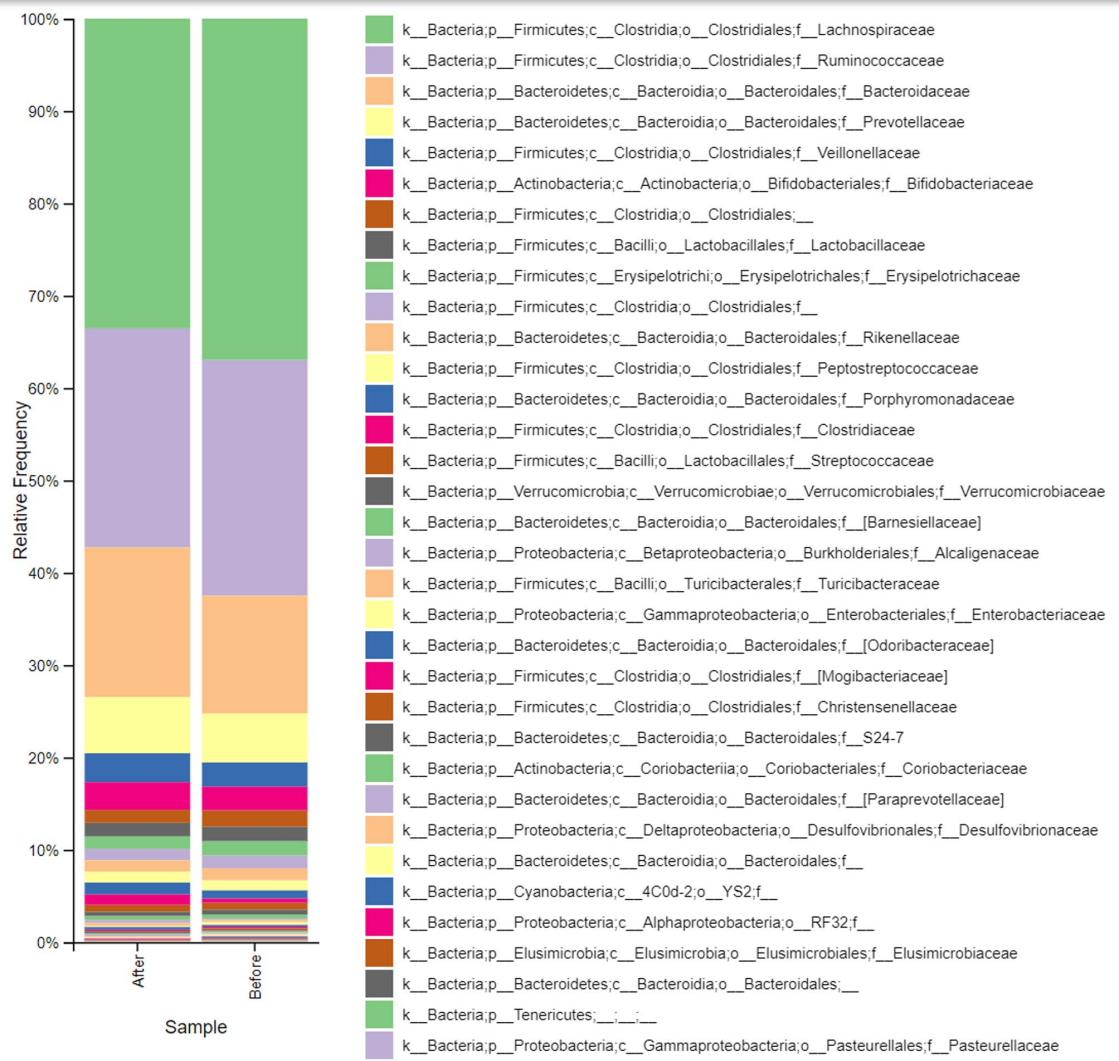
Relative bacterial abundance at the family level in 40 fecal samples collected before and after water kefir consumption. All pre-consumption and post-consumption samples were aggregated and presented as summary bars to improve visualization and facilitate interpretation of the data.

Analysis of the relative abundance in samples collected before the start of consumption revealed that the predominant bacterial phyla were Firmicutes and Bacteroidetes, followed by Actinobacteria (Fig. 2). During the intervention, the relative abundance of Firmicutes decreased by 6.5%, while Bacteroidetes and Actinobacteria increased by 21.6% and 14.8%, respectively. At the family level, *Bacteroidaceae* exhibited the most pronounced change, with a 27.5% increase, while *Lachnospiraceae* and *Ruminococcaceae* decreased by 9.2% and 7.0%, respectively (Fig. 3). At the species level, significant (*p* ≤ 0.05) increases were observed for *Blautia* spp. (16.5%) and *Roseburia faecis* (12.7%).

#### Alpha and beta diversity

**Fig. 4:**
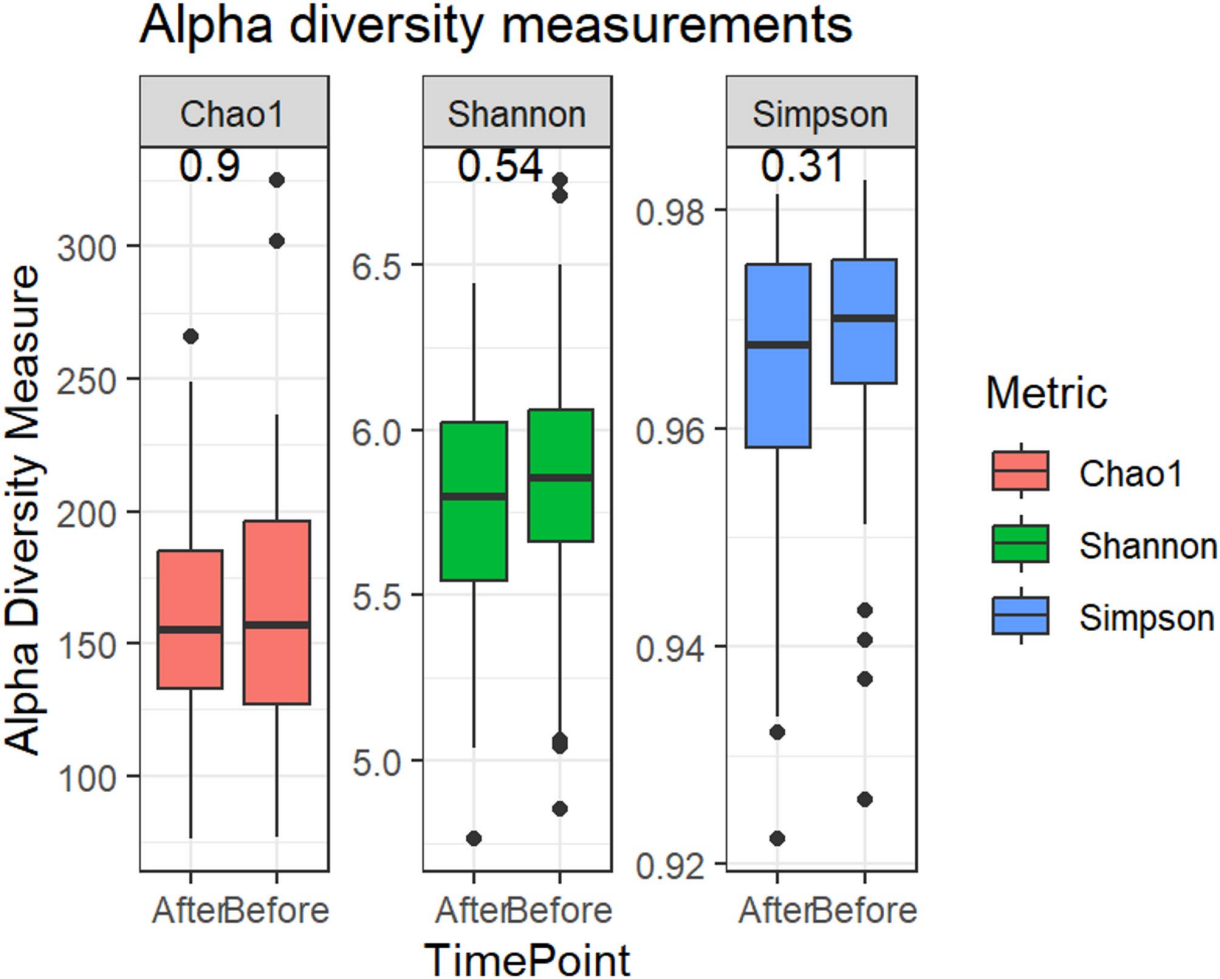
*α*-diversity measurements for fecal microbiota, using Chao1, Shannon, and Simpson indices before and after the intervention. The *p-*values were obtained from the Wilcoxon signed-rank test.

Alpha diversity, reflecting the richness and evenness of species within each sample, was calculated using the Chao1, Shannon, and Simpson indexes based on amplicon sequence variants (ASVs). No significant changes were observed following the 14-day water kefir intervention (Fig. 4). Specifically, the Chao1 index, which estimates species richness, produced a *p-*value of 0.90. The Shannon index, which reflects both richness and evenness of the microbial community, showed a *p-*value of 0.54. Similarly, the Simpson index, which places greater emphasis on dominant species and evenness, yielded a *p-*value of 0.31.

**Fig. 5:**
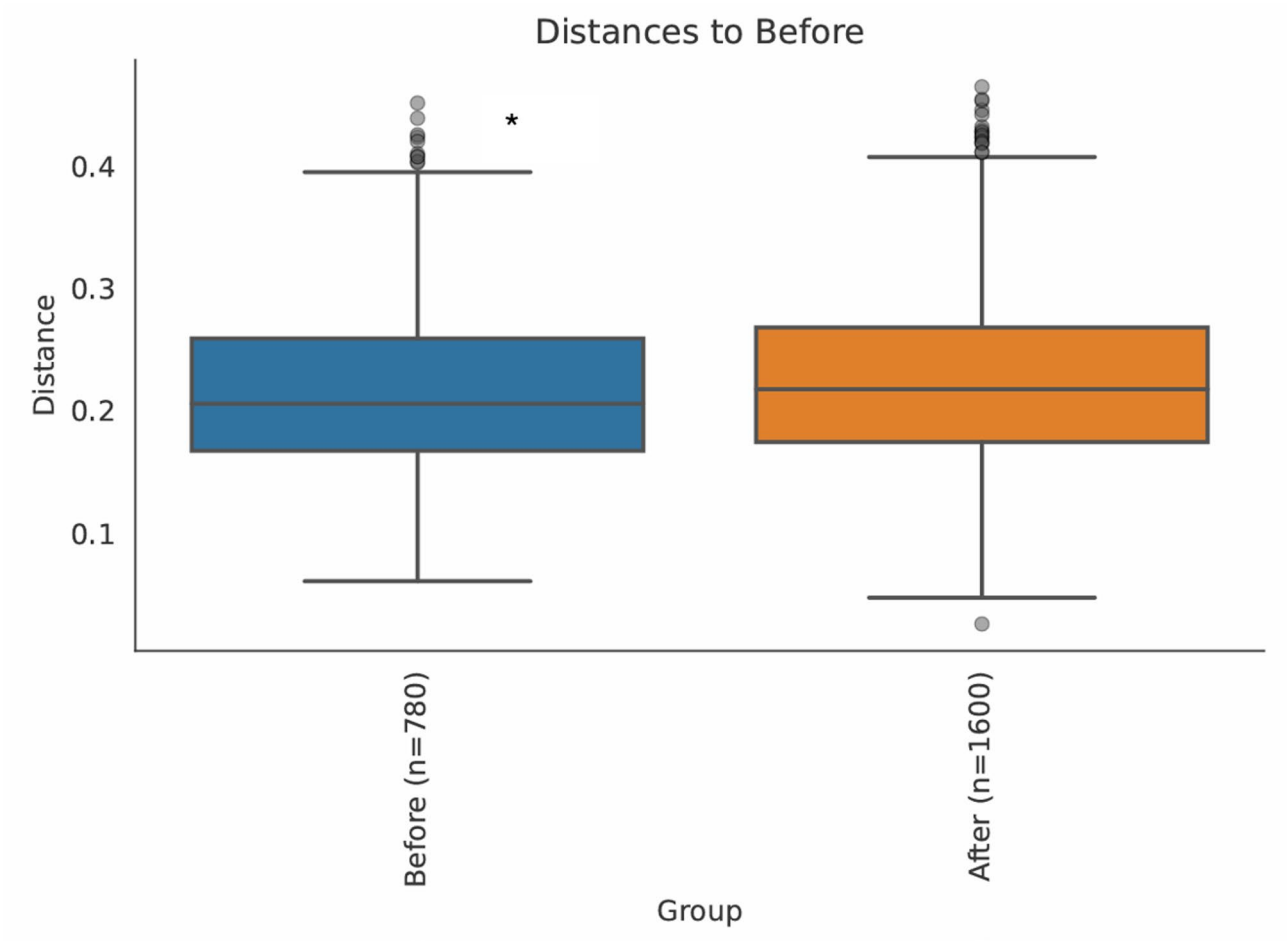
*β*-diversity comparison of microbial communities before and after the intervention using Weighted UniFrac distance. The boxplot shows the distribution of pairwise distances within and between the two groups. ANOSIM analysis revealed a statistically significant difference between groups (*p* = 0.025). *Indicates *p* = 0.025 compared to after the intervention.

Beta diversity, assessing differences in microbial community composition between samples, was evaluated using ANOSIM analysis. A statistically significant shift was detected between samples collected before and after the intervention (*p* = 0.025). Pairwise distance analysis indicated that the magnitude of this difference was modest (Fig. 5).

#### Changes in amplicon sequence variants (ASVs) over time

**Fig. 6:**
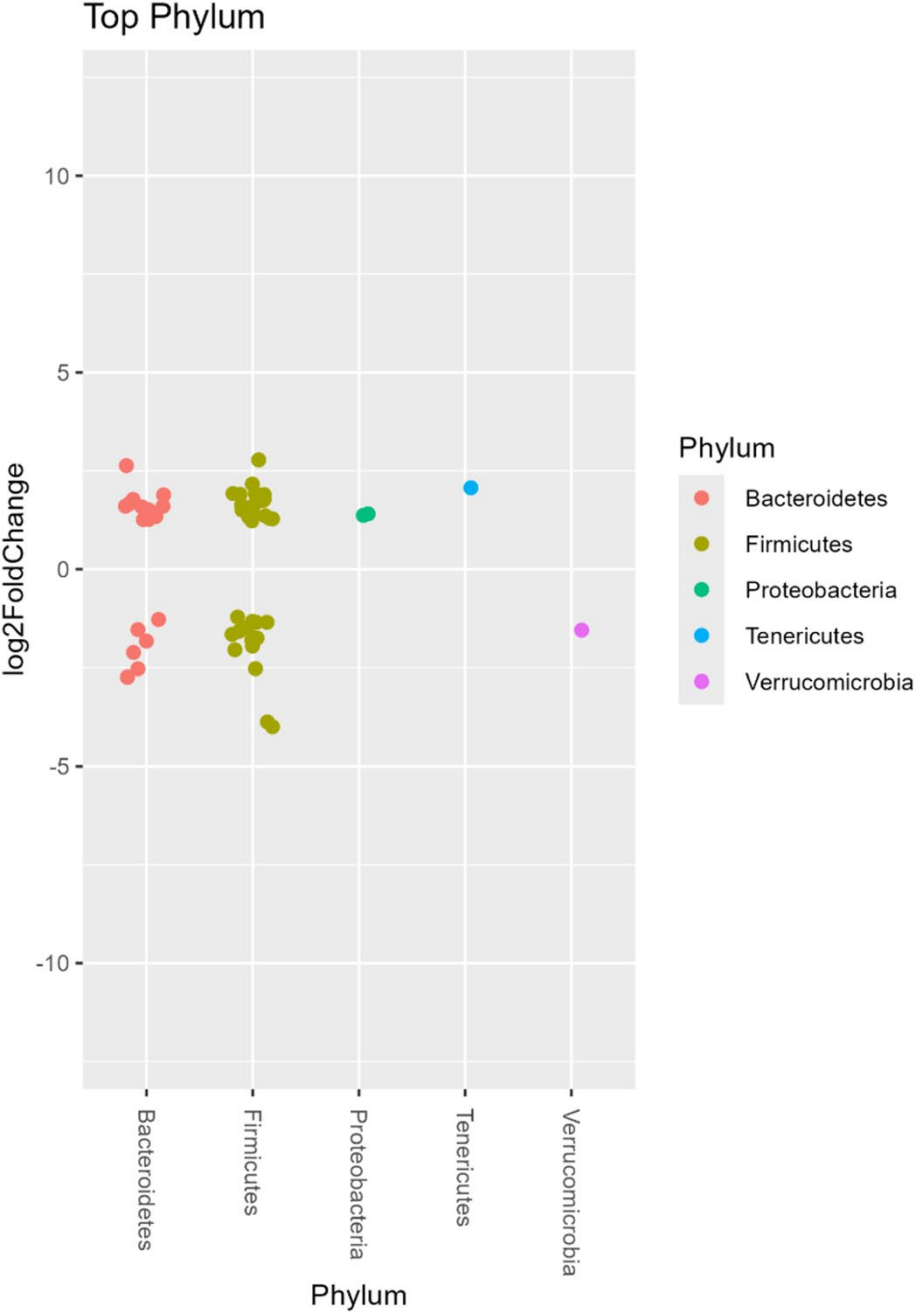
Differential abundance of microbial phyla before and after the intervention. Each point represents an amplicon sequence variant (ASV), grouped by phylum along the x-axis, and the y-axis indicates the log2 fold change in abundance. Points above zero represent increased abundance after the intervention, whereas points below zero represent decreased abundance. Multiple points within the same phylum reflect variability in the response of individual ASVs. Analysis was conducted using DESeq2 with significance set at adjusted *p* < 0.01.

Changes in the relative abundance of amplicon sequence variants (ASVs) at the phylum level in fecal samples over the 14-day water kefir consumption period are presented in Fig. 6. Proteobacteria and Tenericutes showed consistent increases over the intervention period, whereas Verrucomicrobia decreased significantly (*p* < 0.01). In contrast, Bacteroidetes and Firmicutes exhibited bidirectional changes, with some ASVs increasing and others decreasing in abundance, reflecting variability at finer taxonomic resolution.

**Fig. 7:**
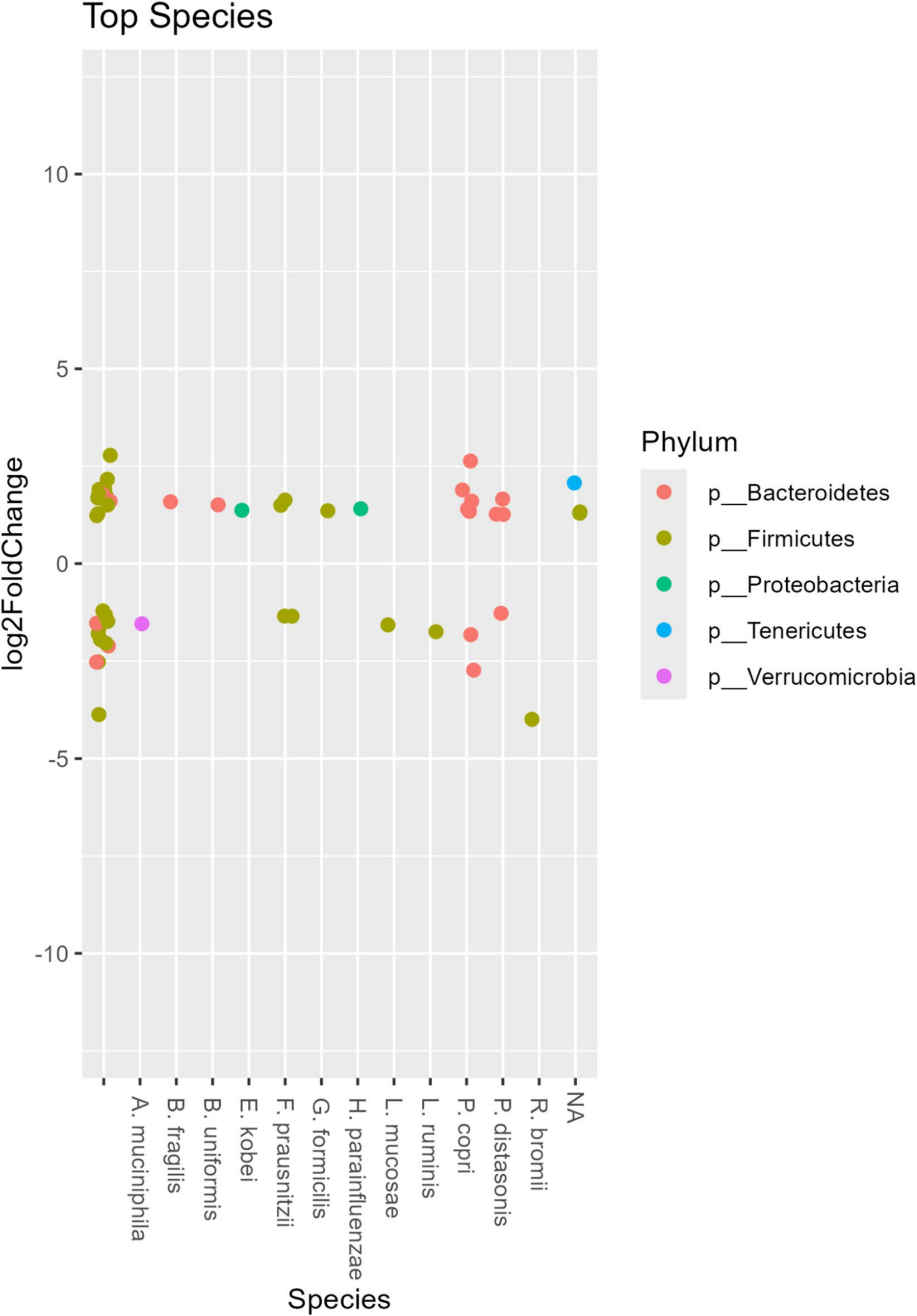
The differential abundance of microbial species between before and after the intervention. Each point represents a species, positioned along the x-axis based on taxonomic classification, and the y-axis depicts the log2 fold change in abundance. Points are color-coded according to their phylum. Analysis was conducted using DESeq2 with significance set at adjusted *p* < 0.01. Positive values indicate an increased abundance after the intervention compared with before, whereas negative values indicate a decrease.

Similarly, changes in the relative abundance of the predominant bacterial species ASVs are illustrated in Fig. 7. Over the intervention period, significant increases were observed in *Bacteroides fragilis*, *Bacteroides uniformis*, *Enterobacter kobei*, *Gemmiger formicilis*, *Haemophilus parainfluenzae*, *Prevotella copri*, *Parabacteroides distasonis*, and *Ruminococcus bromii* ASVs. Conversely, *Akkermansia muciniphila*, *Limosilactobacillus mucosae*, *Lactobacillus ruminis*, and *Ruminococcus bromii* ASVs showed decreased relative abundance (*p* < 0.01).

### Comparative analysis of shared and distinct genera in water kefir and gut microbiota of participants

**Fig. 8:**
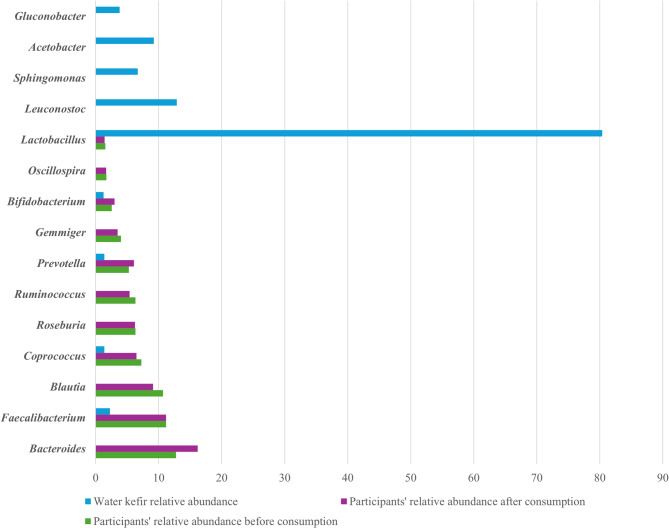
Relative abundance of overlapping and unique bacterial genera in participants’ gut microbiota and the water kefir. Each genus is represented by three bars: one showing its abundance in water kefir, and two showing its abundance in the gut microbiota before and after the intervention.

The clustered bar chart on Fig. 8 illustrates the relative abundance above detection limit of bacterial genera found in water kefir and in participants’ gut microbiota. Each genus is represented by three bars: one for the water kefir, and two for the gut microbiota pre- and post-intervention—allowing for a visual comparison of shared and unique genera.

The results highlight several overlapping genera including *Lactobacillus*, *Bifidobacterium*, *Prevotella*,* Coprococcus*, and *Faecalibacterium*, which were present in both microbiotas, at different abundances. For these genera, the level of *Bifidobacterium* and *Prevotella* increased, meanwhile *Coprococcus* decreased and *Lactobacillus* and *Faecalibacterium* remained stable when comparing before and after consumption. Notably, *Lactobacillus* and *Leuconostoc* were dominant in the water kefir, but were absent or found at lower levels in the participants’ microbiota. Conversely, genera including *Oscillospira*, *Gemmiger*, *Ruminococcus*, *Roseburia*, *Blautia*, and *Bacteroides* were present at varying levels in the participants’ gut microbiota, but were not detected in the water kefir. Of these genera, the level of *Gemmiger*,* Ruminococcus*, and *Blautia* decreased, meanwhile *Roseburia* remained stable and *Bacteroides* increased post intervention.

## Discussion

Fermented foods are increasingly recognized for their influence on gut health, partly due to their content of live microorganisms with potential probiotic properties. However, few intervention trials have addressed the impact of non-dairy fermented beverages such as water kefir. In this study, we examined how daily consumption of water kefir influences gut microbiota composition in healthy adults.

Adherence to daily water kefir consumption was maintained by most participants; however, 21.1% missed one or two days during the intervention period. Gastrointestinal responses varied among participants. While some reported increased flatulence (32%) and bloating (24%), others experienced a reduction in abdominal pain (28%). The majority (66%) reported no noticeable change in gastrointestinal symptoms, and only a small proportion (3%) reported transient stomach pain. Mild gastrointestinal discomfort, including flatulence and bloating, is commonly observed when introducing live microorganisms and is generally considered a temporary response as the gut microbiota adapts to dietary changes^21,22^.

In addition to self-reported outcomes, the impact of water kefir intake on gut microbial composition was assessed using high-resolution taxonomic analysis. At the family level, *Bacteroidaceae* showed the most notable increase (27.5%), whereas *Lachnospiraceae* and *Ruminococcaceae* declined by 9.2% and 7.0%, respectively (Fig. 3). Interestingly, although the overall abundance of *Lachnospiraceae* decreased, specific taxa within this family, including *Blautia* spp. and *Roseburia faecis*, increased significantly. This highlights the complexity of microbial community dynamics, where trends at higher taxonomic levels may mask divergent patterns at finer resolution.

*Blautia* and *Roseburia* species are widely recognized as producers of short-chain fatty acids (SCFAs), particularly butyrate, a microbial metabolite which plays an important role in gut health. These genera have been associated with healthy gut microbiota profile and play key roles in modulating gut inflammation, supporting the immune system and promoting gut barrier integrity, although, their specific roles may vary depending on species and strain^23–27^. These findings are consistent with previous in vitro and animal studies suggesting that water kefir may promote SCFA production and promote the growth of bacterial taxa associated with beneficial metabolic functions^28^.

Members of the *Lachnospiraceae* family also play a central role in the degradation of complex plant-derived polysaccharides, such as cellulose and hemicellulose, which are otherwise indigestible by the human host. Several species within this family, including *Blautia* spp., and *Roseburia faecis*, have been associated with dietary intake of plant-based proteins, and fibre^29^. Water kefir, containing fermentable substrates and microbial metabolites, may provide conditions that support the growth of such taxa.

To complement taxonomic findings, diversity analyses were conducted. Although *α*-diversity metrics showed no significant changes following the intervention (Fig. 4), this indicates that overall microbial richness and evenness remained stable within individuals. In contrast, *β*-diversity differed significantly between pre- and post-intervention samples (Fig. 5), indicating that the composition of the microbial community differed between timepoints. Together, these findings suggest that water kefir consumption influenced the relative abundance of specific taxa without substantially altering overall microbial diversity.

These observations are consistent with previous human probiotic and dietary intervention studies of similar cohort size, in which overall diversity often remains stable despite shifts in specific microbial taxa or host-related outcomes^30,31^. Similar findings have been reported in both infants and adults, where beneficial effects may occur without major changes in overall diversity^32,33^. Notably, the statistically significant shift in *β*-diversity observed in this study suggests that water kefir consumption influenced the overall composition of the microbiota between timepoints, although the effect size was modest. Together, these findings underscore both the resilience and the dynamic potential of the gut microbiota in response to the intervention.

The relatively short duration of the intervention (14 days) represents an important limitation and indicates that the findings should be interpreted as reflecting short-term microbiota responses. While significant shifts in *β*-diversity were detected, it remains unclear whether these changes are transient or would persist with continued consumption. Previous studies have shown that short-term dietary interventions are known to induce rapid changes in gut microbiota composition^34,35^, whereas long-term dietary patterns are more strongly associated with sustained diversity and functional characteristics, highlighting both the adaptability and resilience of the gut microbiome^36,37^. Longer-term consumption of water kefir may lead to more stable or pronounced changes, although this requires further investigation.

Differential abundance analysis further demonstrated that, although overall diversity remained stable, the composition of the microbiota shifted, with certain taxa increasing and others decreasing in response to the intervention (Figs. 6 and 7). The relative abundance of Actinobacteria and Bacteroidetes increased, whereas Firmicutes decreased. The resulting reduction in the Firmicutes-to-Bacteroidetes ratio is consistent with previous observations associated with metabolic health, although such relationships remain complex and context-dependent^38^. The magnitude of these changes is reflected in relative abundance differences and log2 fold changes derived from DESeq2 analysis.

While several taxa identified in this study are commonly associated with beneficial functions, such interpretations should be made with caution, as microbial effects are often context-dependent and influenced by host and environmental factors. Notably, increases were observed in taxa such as *Bacteroides fragilis* and *Bacteroides uniformis*, which are a part of the normal commensal gut microbiota and have been associated with immune modulation and intestinal homeostasis^39,40^. However, other taxa, including *Enterobacter kobei* and *Haemophilus parainfluenzae*, may act as opportunistic pathogens under certain conditions. Their presence in healthy individuals is not uncommon, and their functional roles likely depend on host and environmental factors^41–43^. The observed reduction in *Akkermansia muciniphila*, a mucin-degrading bacterium frequently associated with gut health and metabolic benefits, warrants careful interpretation. Although often considered beneficial, its role is complex and may vary depending on host characteristics, baseline microbiota composition, and strain-level differences. Therefore, the decrease observed in this study should not be interpreted as inherently detrimental^44^.

The bidirectional changes observed in taxa such as *Faecalibacterium prausnitzii*, *Prevotella copri*, and *Parabacteroides distasonis* likely reflect strain-level variability and dietary influences. These species encompass diverse strains with distinct functional roles, and their effects may vary depending on context^45–48^.

In the comparison of overlapping and unique genera between the water kefir and the participants’ gut microbiota, the results indicate that while certain genera present in the water kefir may transiently colonize or influence the gut microbiome, others remain exclusive to either environment. Observed differences in relative abundance likely reflect selective pressures within the gut ecosystem and the transient nature of microbial strains introduced through kefir consumption.

Our findings suggest that while the overall representation of *Blautia* within the community may shift, certain beneficial species within the genus may be promoted by water kefir intake. Among the overlapping genera, *Bifidobacterium* and *Prevotella* increased in relative abundance in the participants’ gut microbiome after consumption, whereas *Coprococcus* decreased and *Faecalibacterium* remained stable (Fig. 8). Several studies have indicated that *Prevotella* is favoured by diets high in plant-based foods or dietary fibre, such as those typically associated with non-western dietary patterns. Contrary to previous observations suggesting an inverse relationship between *Prevotella* and *Bacteroides*, with high *P. copri* abundance typically associated with reduced levels of *Bacteroides*, our study found a parallel increase in both genera/species following water kefir consumption. The concurrent increase in genera such as *Bacteroides* and *Prevotella* may suggest a potential probiotic-like effect. However, further controlled studies are required to confirm functional and clinical relevance^47^.

Despite removal of kefir grains prior to consumption, the observed increase in saccharolytic and SCFA-producing taxa suggests that soluble microbial metabolites, such as exopolysaccharides, which are produced during fermentation and remain in the final beverage, may influence the gut microbiota^49^. Although small amounts of plant-derived ingredients, such as rosehip powder and dried apricot, were used during fermentation, their contribution to dietary fibre intake is likely negligible. The rosehip powder was added at a low concentration and largely removed by filtration, and participants were instructed not to consume the apricot.

Dietary intake represents an important confounding factor in microbiome studies. Participants were instructed to maintain their habitual diet; however, dietary intake was not standardized or recorded in detail. Variations in fibre and carbohydrate intake may therefore have influenced observed microbial changes, particularly in taxa such as *Prevotella* and *Bacteroides* which are known to respond to dietary composition.

While allowing participants to follow their usual diet enhances ecological validity, it may also contribute to inter-individual variability and limits the ability to attribute observed microbiota changes solely to the intervention. Future studies incorporating dietary monitoring or controlled dietary conditions would provide a more precise understanding of the relationship between water kefir consumption and gut microbiota modulation.

## Study limitations

The findings of this study should be interpreted primarily as microbiological and exploratory in nature. Although changes in gut microbiota composition were observed, their clinical relevance remains unclear. Gastrointestinal responses to the intervention were variable among participants, and no functional or metabolic outcomes were assessed. Therefore, the observed microbial shifts should not be directly interpreted as indicators of improved health outcomes. Future studies incorporating clinical endpoints, metabolic markers, and functional analyses are needed to better understand the potential health implications of water kefir consumption.

Gastrointestinal symptoms were assessed using a self-reported questionnaire that was not a formally validated clinical instrument. Although the questionnaire was adapted from previously validated tools, the lack of formal validation may limit the reliability and comparability of the symptom data.

This study was designed as an exploratory investigation, and no formal power calculation was performed prior to participant enrolment. As such, the study may be underpowered to detect smaller effects, and the findings should be interpreted with caution.

The relatively small sample size (*n* = 40) and the wide age range of participants (18–65 years) may limit the generalizability of the results and contribute to inter-individual variability in microbiota composition and responses to the intervention. Inter-individual variability is an inherent feature of human microbiome studies and may have influenced both baseline microbial composition and observed responses. Factors such as age, habitual diet, geographical background, and individual microbiota profiles likely contributed to the heterogeneity observed, including bidirectional changes in certain taxa and the modest effect size detected in *β*-diversity.

The within-subject (pre–post) design partially mitigates inter-individual variability by allowing each participant to serve as their own control; however, the absence of a separate control group could be a limitation. External factors such as temporal variation, habitual dietary intake, and placebo effects cannot be excluded, limiting the ability to attribute observed changes specifically to water kefir consumption.

Participants were instructed to maintain their habitual diet to reflect real-life consumption patterns. While this enhances ecological validity, it may also introduce additional variability, as dietary intake was not standardized or recorded in detail. Variations in fibre and carbohydrate intake may therefore have influenced the observed changes in microbial taxa.

Finally, the home-based preparation of water kefir may have introduced variability in fermentation conditions, such as temperature, duration, and hygiene practices. Although standardized ingredients, equipment, and preparation protocols were provided, some variation in the microbial composition of the kefir cannot be excluded, which may have contributed to differences in individual responses. This approach was chosen to reflect real-life consumption conditions, as water kefir is commonly prepared at home and is not yet widely commercially available in many countries. Future studies employing controlled or centralized preparation methods may help to reduce this variability.

## Data Availability

The raw sequencing data have been submitted to the Sequence Read Archive (SRA) of NCBI, accessible via (https://www.ncbi.nlm.nih.gov/bioproject/PRJNA1284092) under the BioProject ID PRJNA1284092.
